# Local Application of Chloroform in Sciatica, Etc.

**Published:** 1851-05

**Authors:** J. H. J. Hook

**Affiliations:** St. Matthews, S. C.


ARTICLE VIII.

On the local application of Chloroform in Sciatica, etc.—By J. H. J. Hook, M.D., St. Matthews, S. C.

Messrs. Editors:—Having noticed in the November number of this Journal, that Chloroform has been applied locally by Prof. Bouisson, for the relief or cure of Orchitis, I will give you the result of my limited experience with the article applied locally for some painful diseases, which, by the by, may be a very common mode of using it, for aught I know. However, the first account of its local application that I recollect to have seen, was in Wood's Quarterly Retrospect for April, 1849, taken from the London Medical Gazette. And now for the first case in which I applied it as a local remedy:

"On the 21st of August, 1849, I was summoned to see a gentleman, fifty years of age, suffering from an exquisite pain in the hip joint, which I concluded was sciatica, as I had treated him for the same painful affection several times during the past five years, though it did not always attack the hip; sometimes attacking the loins, lumbago, and at other times the knee. During these attacks the pulse would be very slightly excited. The general health was good. He had suffered severely in his youth from white swelling, and is lame from a permanent injury of the knee joint, caused by that disease. The treatment pursued for forty-eight hours before resorting to the Chloroform, consisted in the application of various counter-irritants, locally, (among them Granville's lotion,) cupping, etc., which usually relieved him. I would then have had recourse to constitutional treatment for the relief of the pain, but I knew how averse he was to every thing of an anodyne nature, and that it would be impossible to induce him to take any. Failing thus to relieve him by the usual means, I determined to try Chloroform locally.

On the morning of the 23d, I applied it over the sacrum and loins, by saturating a piece of flannel with it, four by six inches, folded four times, and confining it to the part with a large towel as I had nothing more suitable at hand. In a few minutes, the patient cried out from pain, excited by the Chloroform, and asked what in the name of heaven I had put on him. I replied to him to be quiet for a while, and that I hoped he would soon be better. In five minutes after these words were uttered, he said that he would be perfectly happy if I could make him feel always as he did at that time; and in ten minutes he was asleep, to the great joy of myself and his family, who had been kept up the two preceding nights, in fruitless efforts to relieve him. I must confess here that as this was my first essay with this agent, I watched him with a great deal of anxiety during his nap, which lasted only thirty minutes. In one hour from the first application the pain began to return, and I applied it the second time with the same happy effect; this nap being much longer than the first. It was applied twice more during the same day by members of the family, on the return of pain, and at night I found him in a great measure relieved; so much so, that no further attendance was required. He was able to attend to his business in a few days, and has had no return of it since, either in the knee, hip, or loins.

"I have used it latterly in two other cases, one of neuralgia in the face, the other a painful affection in the left side of a maiden lady, about thirty-five years of age, whose general health is good, with the exception of this pain. There is no excitement in the system at the time the pain appears most violent. She has been repeatedly blistered, cupped, etc., without much benefit. The Chloroform in these cases procures almost immediate relief."—*Charleston Med. Jour.*

